# Supplementary material for: A unique binding mode of Nek2A to the APC/C allows its ubiquitination during prometaphase
Source: EMBO Rep. 2020 Apr 19;21(6):e49831. doi: 10.15252/embr.201949831 (PMC7271329; doi:10.15252/embr.201949831)
Supplement: Supplementary file 1 — Appendix [file EMBR-21-e49831-s001.pdf]

Appendix for

A unique binding mode of Nek2A to the APC/C allows its ubiquitination during prometaphase.

Claudio Alfieri[1,2,3], Thomas Tischer[1] and David Barford[1]

[1]MRC Laboratory of Molecular Biology, Francis Crick Avenue, Cambridge, CB2 0QH, UK.

[2]Current address: Institute of Cancer Research, 237 Fulham Road, London, SW3 6JB, UK.

[3]Correspondence: Claudio.alfieri@icr.ac.uk

Table of contents

Appendix Figure S1: Cyclin A and Nek2A degradation data in HEK cells. *page 2*

Appendix Figure S2: Effect of mutations in the APC/C MR pocket 2 on Ube2S-dependent chain elongation, and analysis of MR and IR tail conservation and binding sites. *page 4*

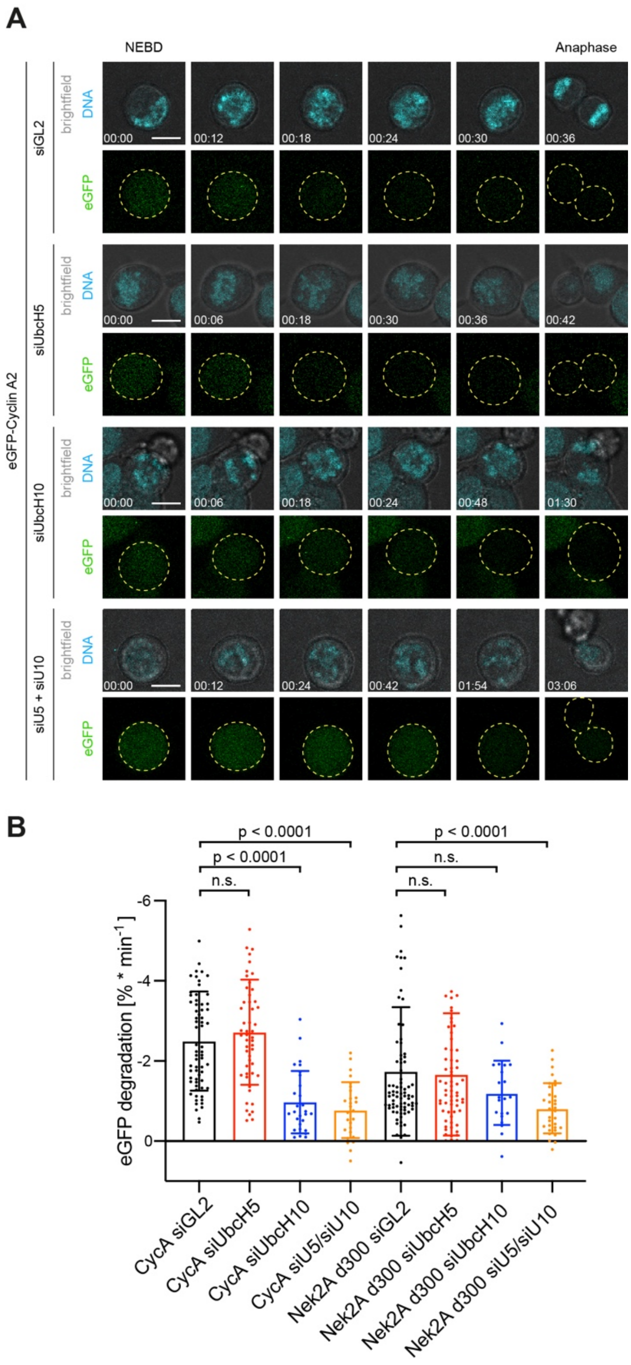

Appendix Figure S1. Cyclin A and Nek2A degradation data in HEK cells.

A. Exemplary still images from time courses between NEBD and anaphase of eGFP-Cyclin A2 destruction in HEK cells. Cells were either treated with siGL2 as control or depleted of the indicated E2 enzymes. The chromosomes are coloured in cyan and eGFP-Cyclin A2 in green, with the outline of the cells are indicated with dashed yellow lines. Time is given as hh:mm. Scale bar 10 µm. See also supplementary movies 5 to 8.

B. Degradation rates of eGFP-Cyclin A2 (left) or eGFP-Nek2A (right) were calculated in cells treated with siGL2 as control or the indicated siRNA. For each cell that was analysed, a linear regression was calculated over the linear part of the normalized degradation curve. The slope of the regression line directly corresponds the decrease of the eGFP signal in percent per minute [% * min^{-1}]. P-values were calculated with a simple ANOVA test followed by Dunnett's multiple comparison. Bar graphs represent mean ± s.d.. The number of cells analysed are N = 66 (Cyclin A2 siGL2), 52 (Cyclin A2 siUbcH5), 28 (Cyclin A2 siUbcH10), 27 (Cyclin A2 siU5/siU10), 80 (Nek2A siGL2), 64 (Nek2A siUbcH5), 21 (Nek2A siUbcH10), 32 (Nek2A siU5/siU10). All data are from at least two biological replicates.

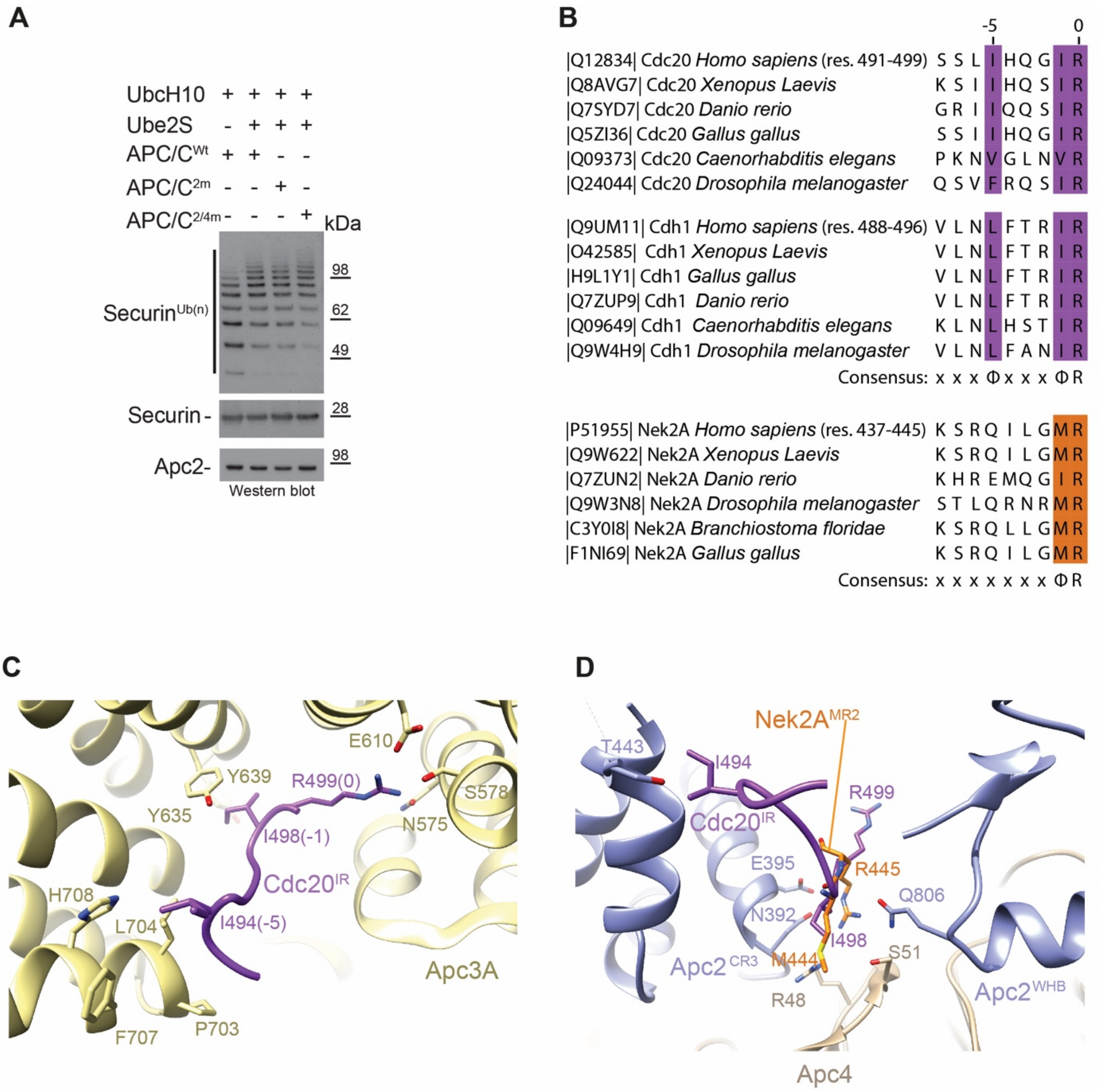

Appendix Figure S2. Effect of mutations of the APC/C MR pocket 2 on Ube2S-dependent ubiquitin chain elongation and analysis of MR and IR tail conservation and binding sites.

- **A.** Securin ubiquitination reactions performed with either APC/C^{Cdc20} wild type (APC/C^{WT}) or mutants, in the presence of Ube2S.
- **B.** Sequence alignment of the IR tail motifs of Cdc20 and Cdh1 coactivators and the MR tail motif of Nek2A. The MR tail of Nek2A differs from the coactivators IR tail motifs because it lacks a hydrophobic residue (ϕ) at position -5.
- **C.** Structure of the Cdc20 IR tail motif (purple) engaged at its binding site the Apc3A (yellow) (PDB ID: 6TLJ) showing that hydrophobic residue at position -5 (Ile494 of Cdc20) contacts Leu704 of Apc3A.

D. Superposition of the Cdc20 IR tail motif (purple) onto the MR tail of Nek2A (orange) bound to the MR pocket 2 comprising Apc2 (light blue) and Apc4 (light brown). In contrast to the IR tail-binding site of Apc3A, the MR pocket 2 lacks the binding site for the IR tail hydrophobic residue (ϕ) at position -5.
