# Supplementary material for: A unique binding mode of Nek2A to the APC/C allows its ubiquitination during prometaphase
Source: EMBO Rep. 2020 Apr 19;21(6):e49831. doi: 10.15252/embr.201949831 (PMC7271329; doi:10.15252/embr.201949831)
Supplement: Supplementary file 2 — Expanded View Figures PDF [file EMBR-21-e49831-s002.pdf]

Expanded View Figures

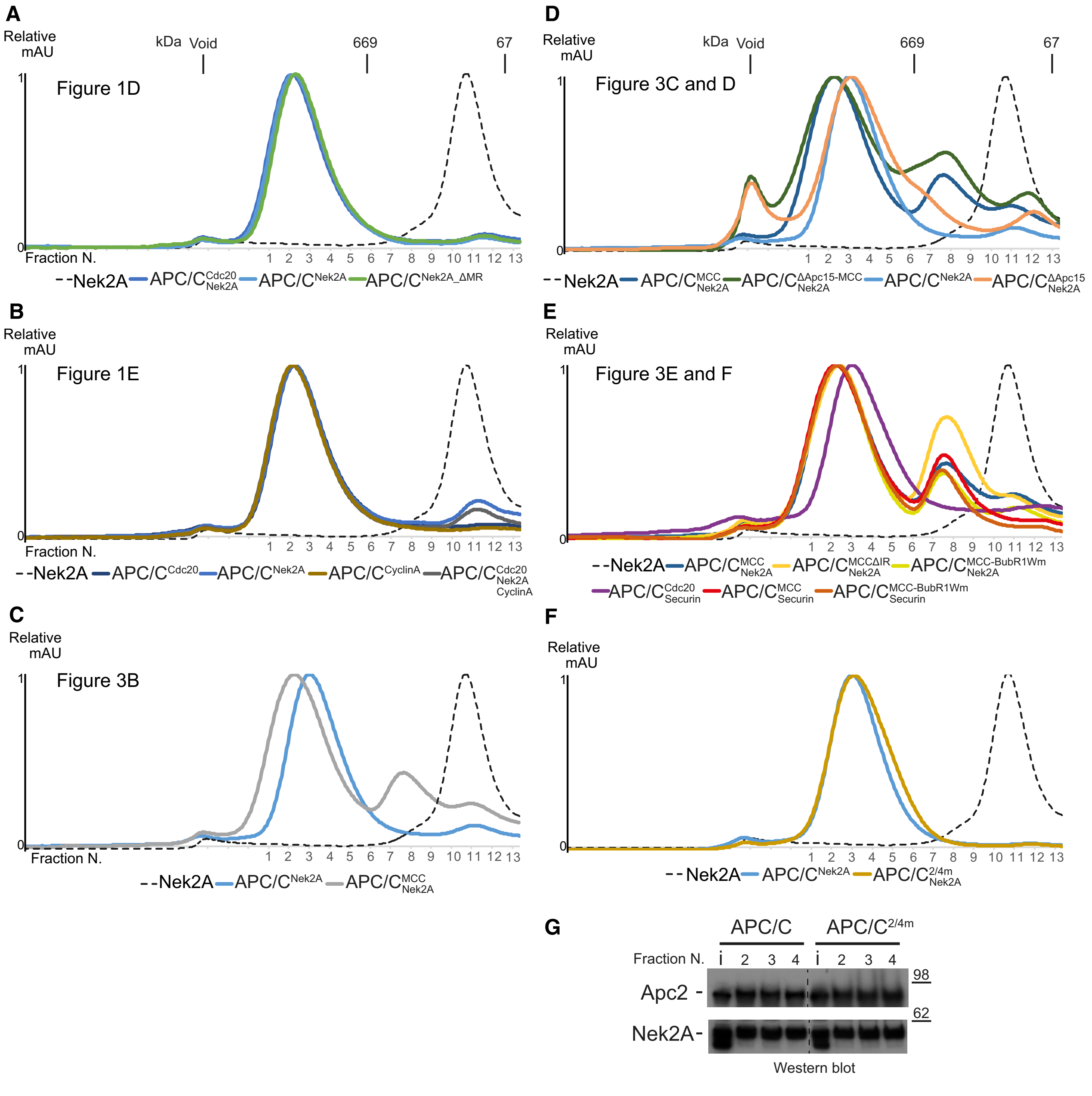

Figure EV1. Binding studies defining the binding mode of Nek2A to the APC/C and APC/C^{MCC} complexes.

A–E Size-exclusion chromatograms of APC/C complexes in this study. The corresponding peak fractions SDS–PAGE gels are shown in the main figures.
F, G Size-exclusion chromatography chromatograms (F) and SDS–PAGE gels (G) of either APC/C wild-type or 2/4 m complexes with Nek2A.

Source data are available online for this figure.

© 2020 The Authors

Figure EV2. APC/C–Nek2A complex sample preparation and cryo-EM analysis.

A SDS–PAGE gels (stained with Coomassie blue) of the gel filtration peak fraction from the APC/C–Nek2A complex preparation used in this study. Western blot against Nek2A confirms the presence of the Nek2A 301–406 construct.
B A typical cryo-EM micrograph of APC/C–Nek2A representative of 6,448 micrographs. Scale bar: 500 Å.
C Gallery of two-dimensional class averages of APC/C^{Nek2A} showing different views representative of 50 two-dimensional classes. Scale bar: 150 Å.
D Fourier shell correlation (FSC) curves are shown for all the cryo-EM reconstructions of APC/C^{Nek2A} determined in this study.
E Fourier shell correlation (FSC) curves are shown for all the cryo-EM reconstructions of APC/C^{MCC} determined in this study using cryo-EM data as published [14].

 © 2020 The Authors

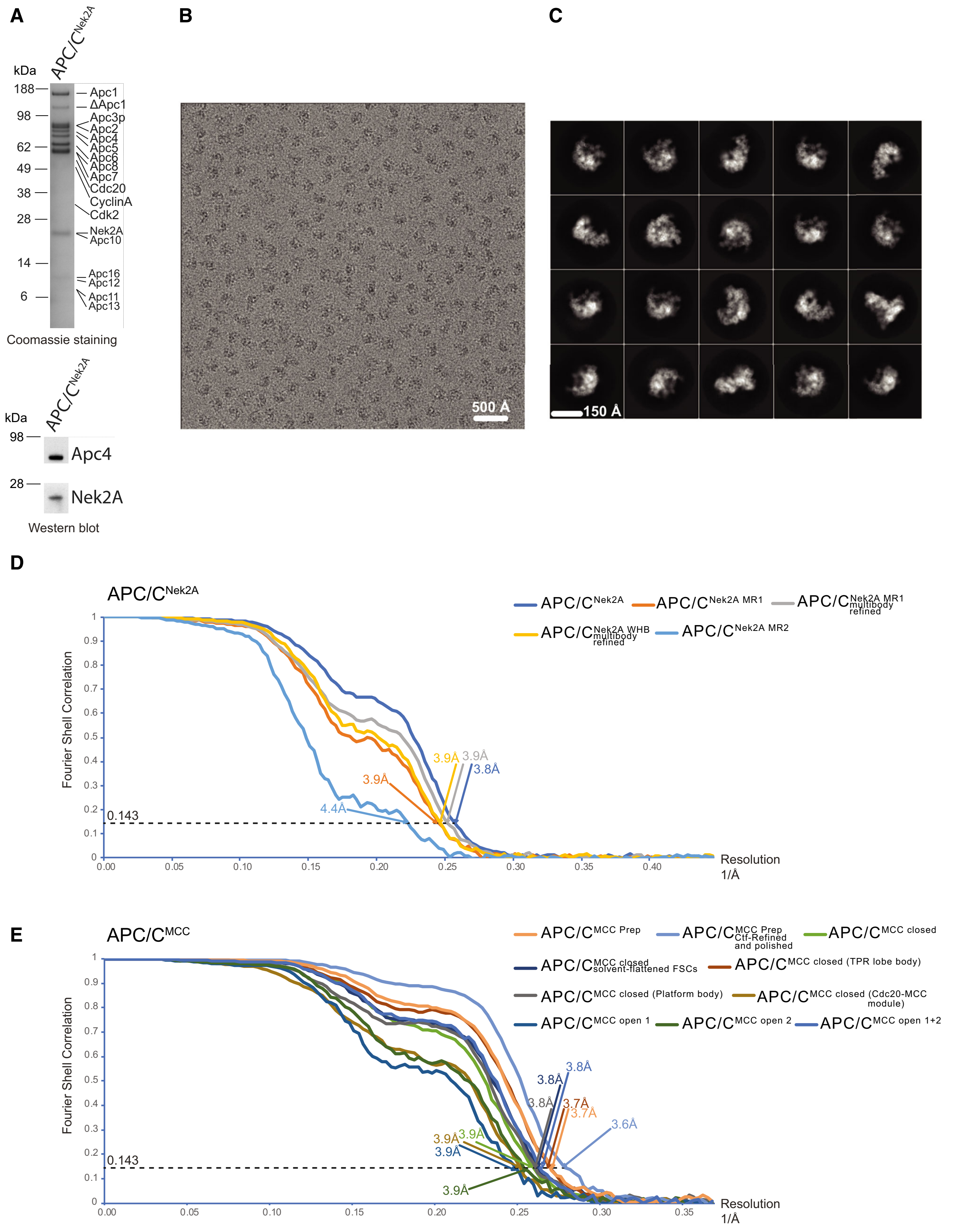
A
APC/C^Nek2A
kDa
188
98
62
49
38
28
14
6
Apc1
ΔApc1
Apc3p
Apc2
Apc4
Apc5
Apc6
Apc8
Apc7
Cdc20
CyclinA
Cdk2
Nek2A
Apc10
Apc16
Apc12
Apc11
Apc13
Coomassie staining
Apc4
Nek2A
Western blot
B
500 Å
C
150 Å
D
APC/C^Nek2A
Fourier Shell Correlation
Resolution 1/Å
0.143
3.9Å
3.8Å
4.4Å
E
APC/C^MCC
3.8Å
3.7Å
3.6Å
3.9Å

Figure EV2.

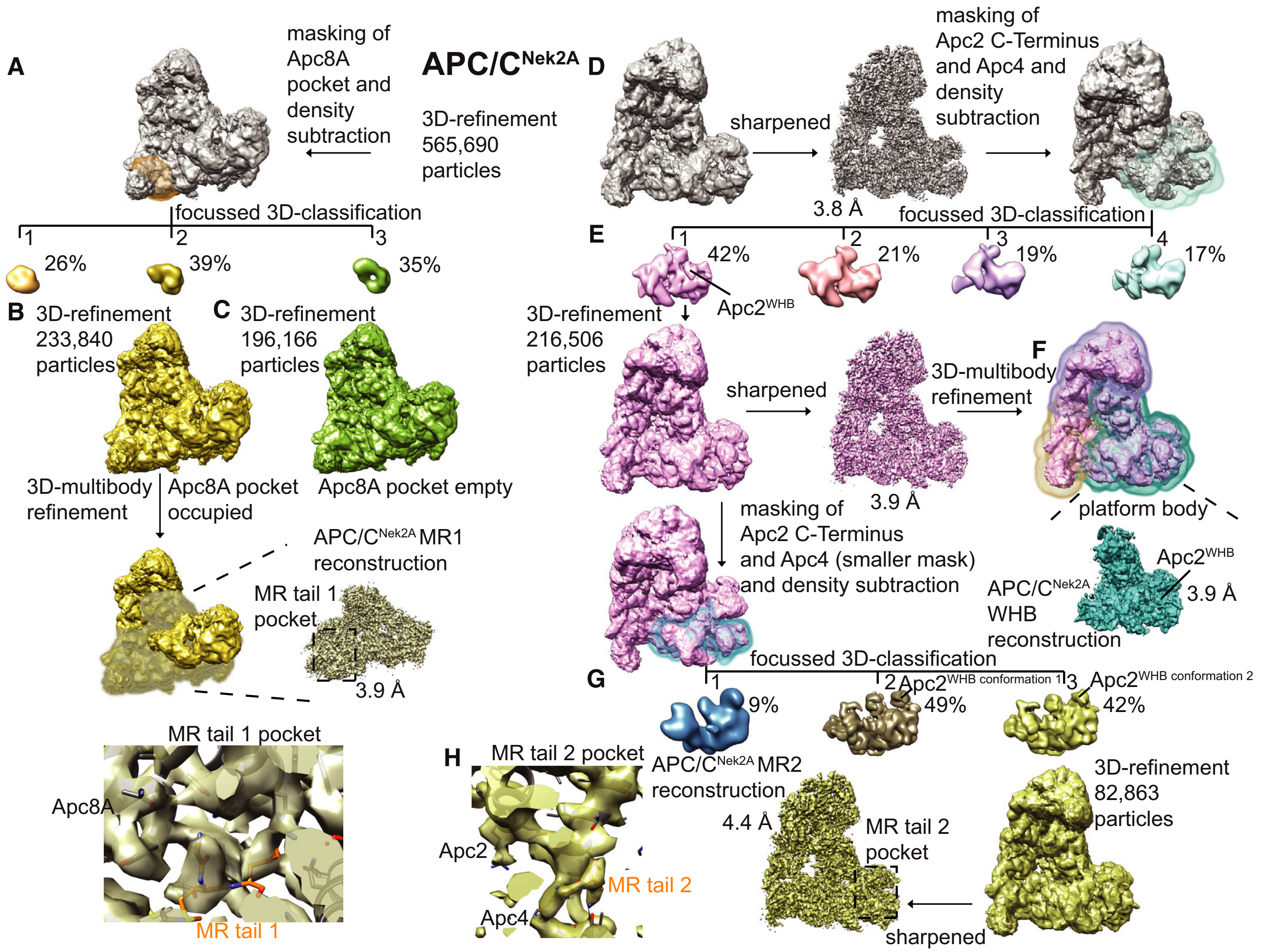

Figure EV3. 3D reconstruction of APC/C–Nek2A complexes.

A–C Workflow for focussed 3D classification and multi-body refinement for 3D reconstruction and refinement of the cryo-EM density for the MR tail 1 binding site on Apc8A.

D–H Workflow for focussed 3D classification and multi-body refinement for 3D reconstruction and refinement of the cryo-EM density for the repositioned APC2^{WHB} domain and for the MR tail 2 binding site on Apc2-Apc4. Classes 2 and 3 shown in (G) differed in the orientation of the APC2^{WHB} domain, and the occupancy of the MR tail 2 was highest in class 3.

 © 2020 The Authors

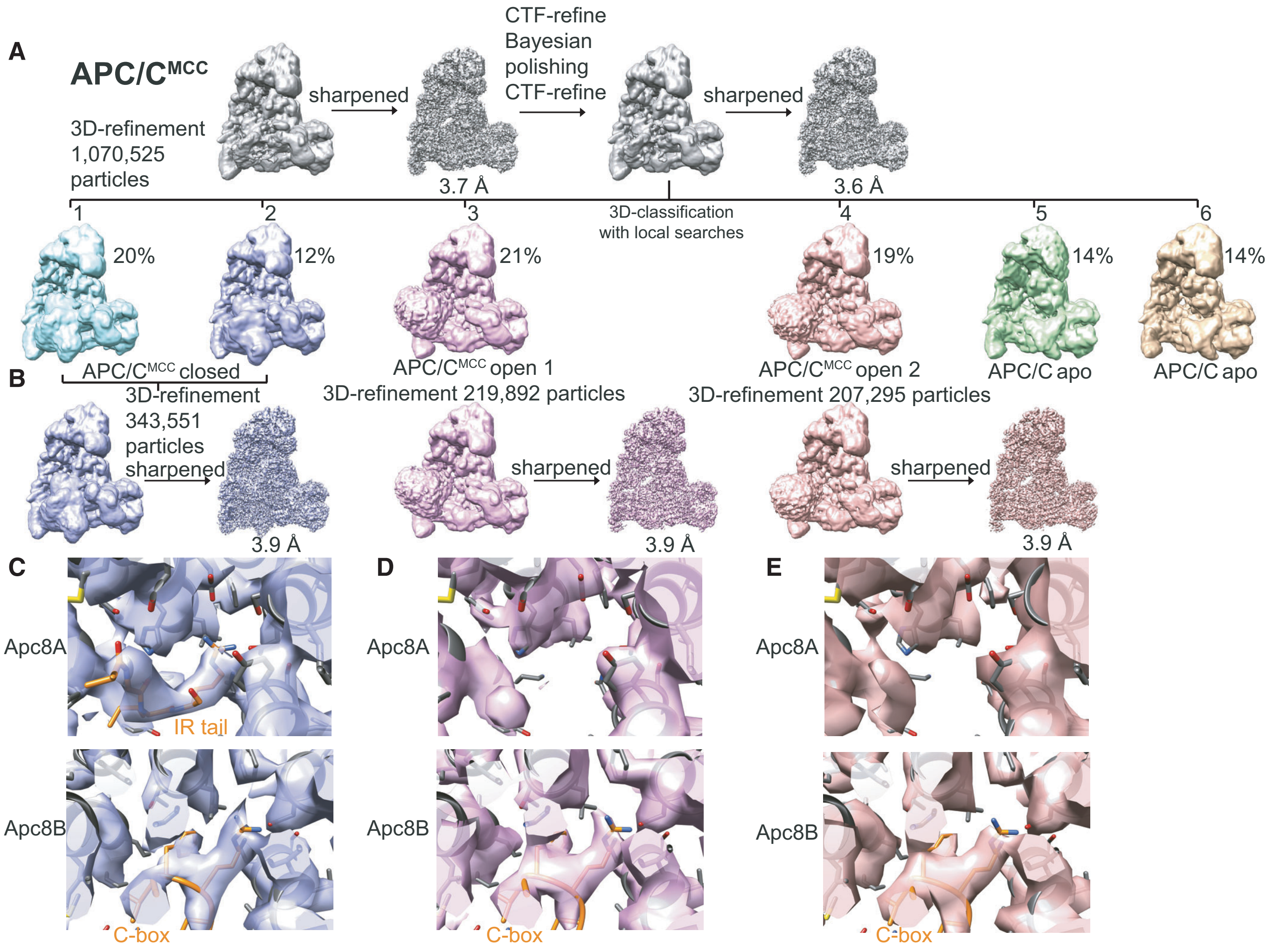

Figure EV4. 3D reconstruction of the APC/C–MCC complex.

A 3D class averages obtained by classification using local searches (see Materials and Methods) are shown for obtaining the APC/C^{MCC} reconstructions.

B Particles from classes 1 and 2 were refined together to obtain the APC/C^{MCC} closed reconstruction. Particles from classes 3 and 4 were separately refined to obtain the APC/C^{MCC} open 1 and 2 reconstructions, respectively. These latter classes were slightly different in the orientation of the Cdc20-MCC module.

C Details of the IR tail and C-box binding site of the APC/C$^{MCC\text{-}closed}$ reconstruction.

D, E (D). Details of the IR tail and C-box binding site of the APC/C$^{MCC\text{-}open-1}$ reconstruction and (E) of the APC/C$^{MCC\text{-}open-2}$ reconstruction. Neither of the two APC/C$^{MCC\text{-}open}$ reconstructions shows clear density for the Cdc20^{MCC} IR tail.

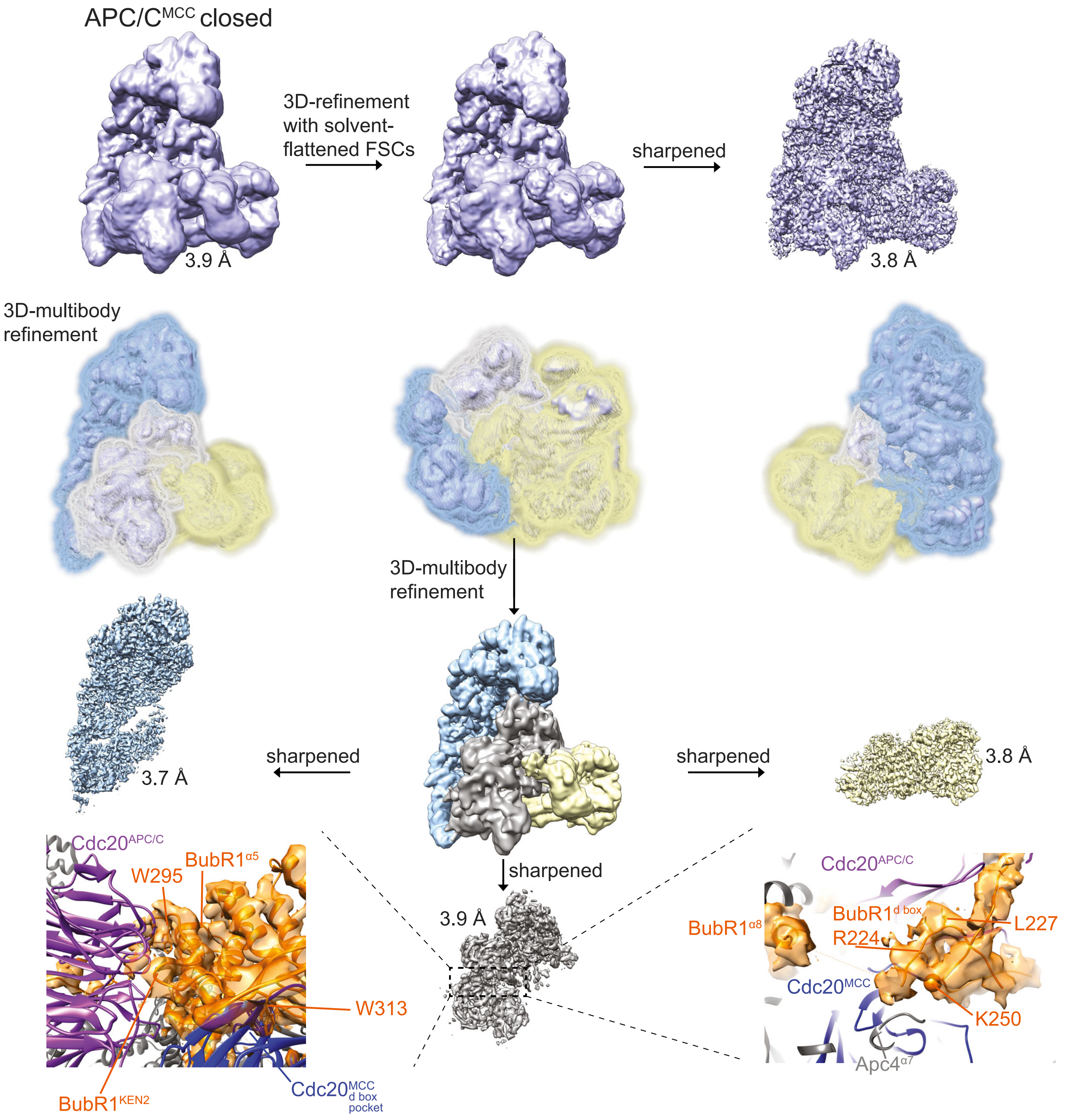

Figure EV5. 3D Multi-body refinement of the APC/C–MCC complex.

3D multi-body refinement strategy for the APC/C$^{MCC\text{-}closed}$ reconstruction (see Materials and Methods). Masks defined for the three APC/C^{MCC} bodies are shown. Details of new BubR1 regions built guided by the improved APC/C^{MCC} cryo-EM map are shown at the bottom. This assignment is consistent with [47–49].

© 2020 The Authors
