# Supplementary figures and images for: A unique binding mode of Nek2A to the APC/C allows its ubiquitination during prometaphase
Source: EMBO Rep. 2020 Apr 19;21(6):e49831. doi: 10.15252/embr.201949831 (PMC7271329; doi:10.15252/embr.201949831)

Appendix Figure S2

A

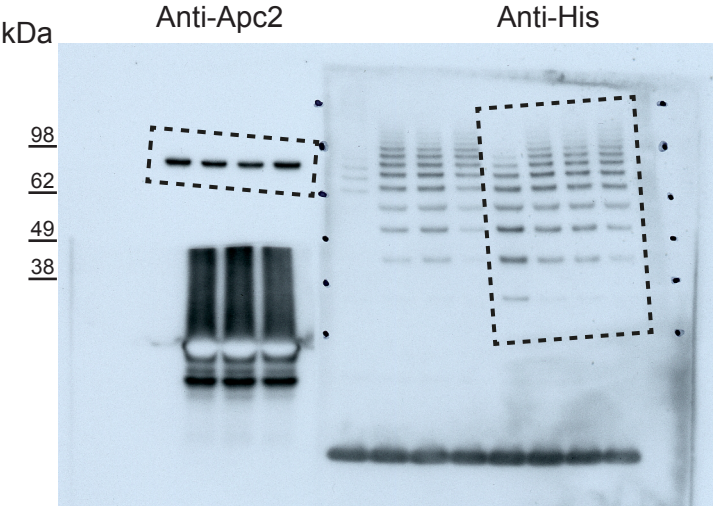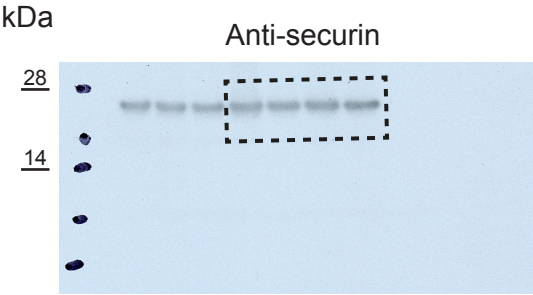

Supplement: Supplementary file 13 — Source Data for Expanded View and Appendix [file EMBR-21-e49831-s017.zip › Source data Blots AppendixFigureS2.pdf]

Figure EV1

G

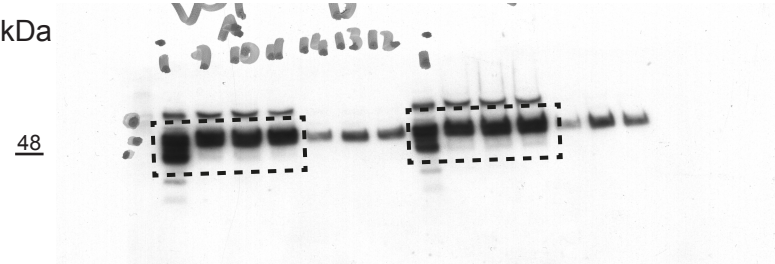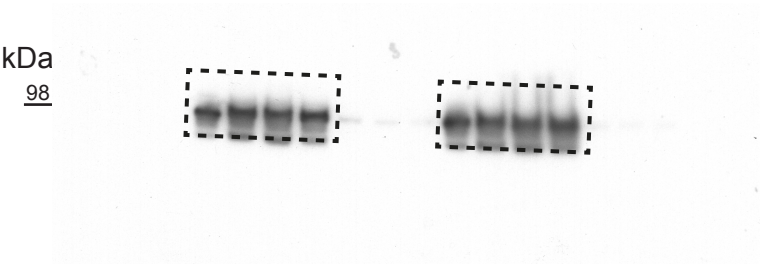

Supplement: Supplementary file 13 — Source Data for Expanded View and Appendix [file EMBR-21-e49831-s017.zip › Source data Blots FigureEV1.pdf]

Figure 1

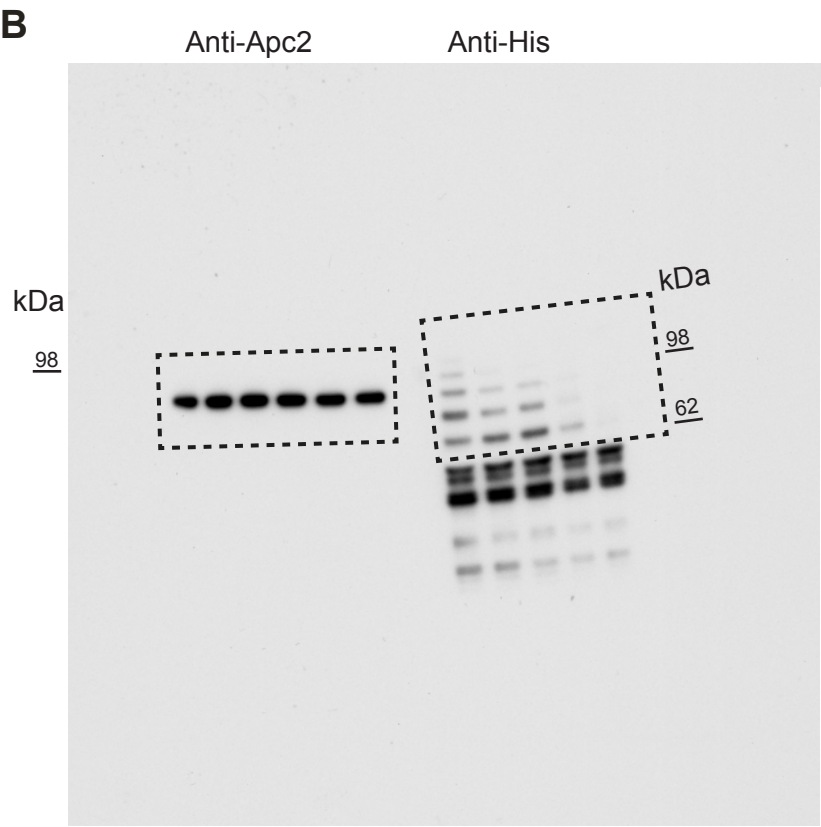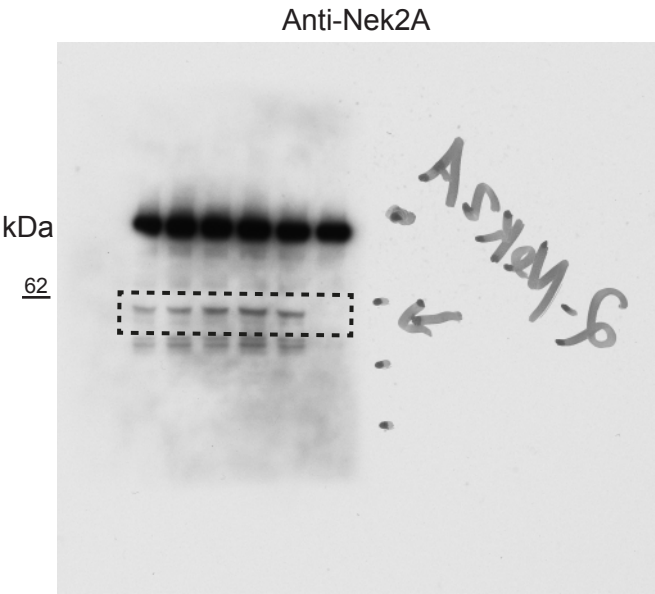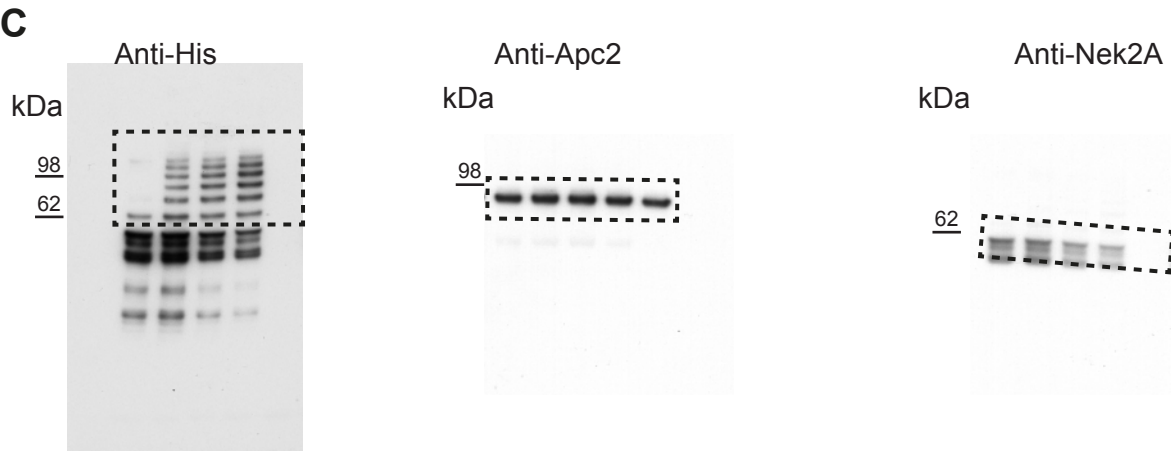

Figure 1

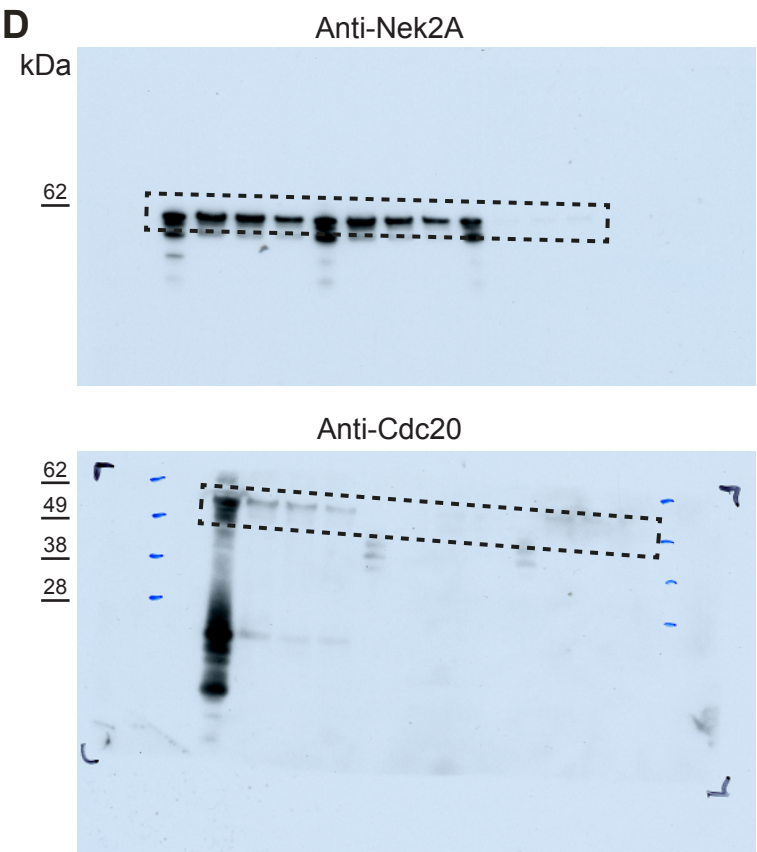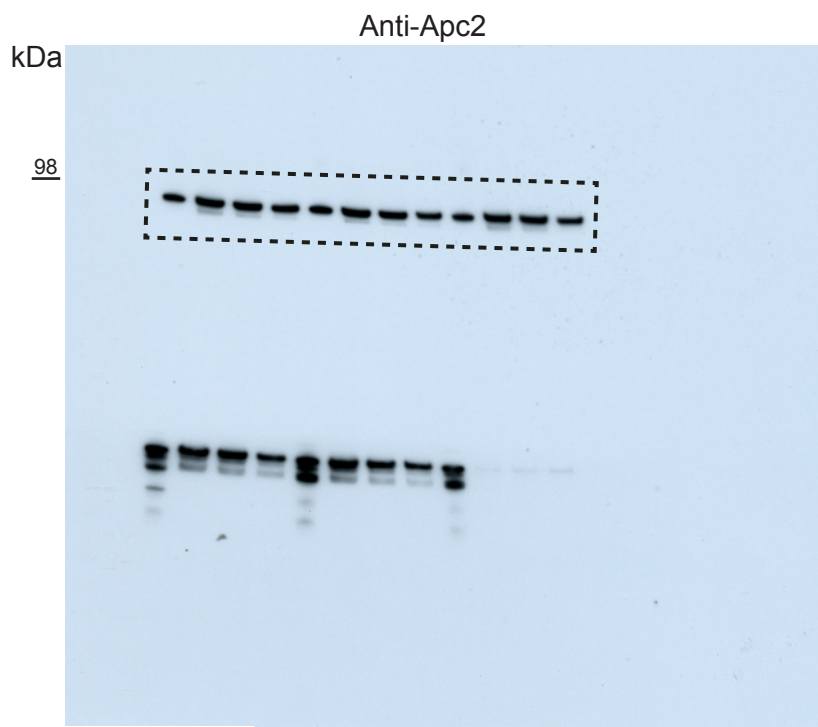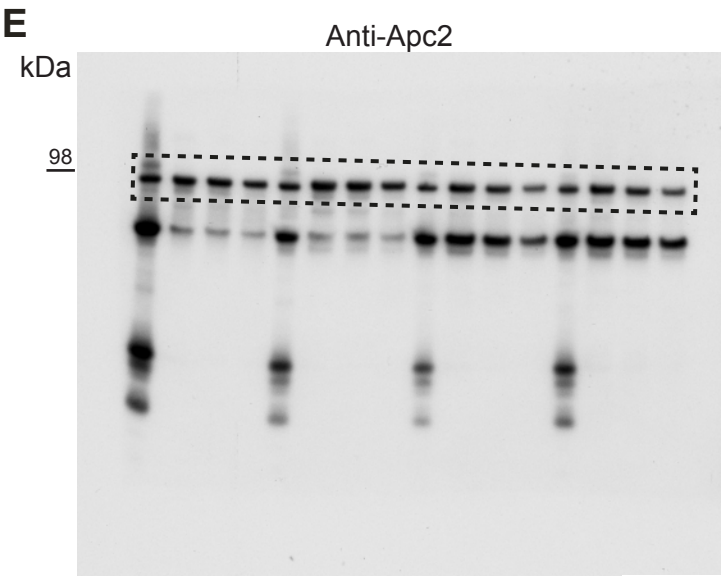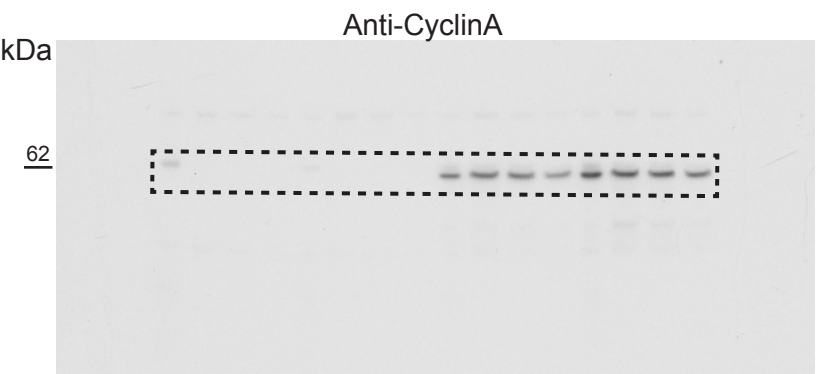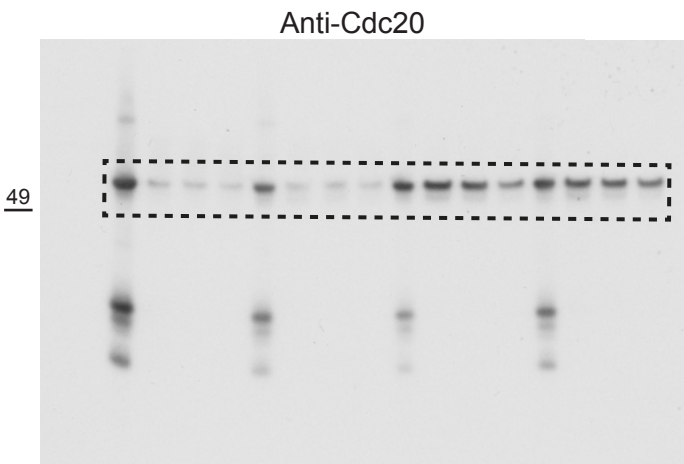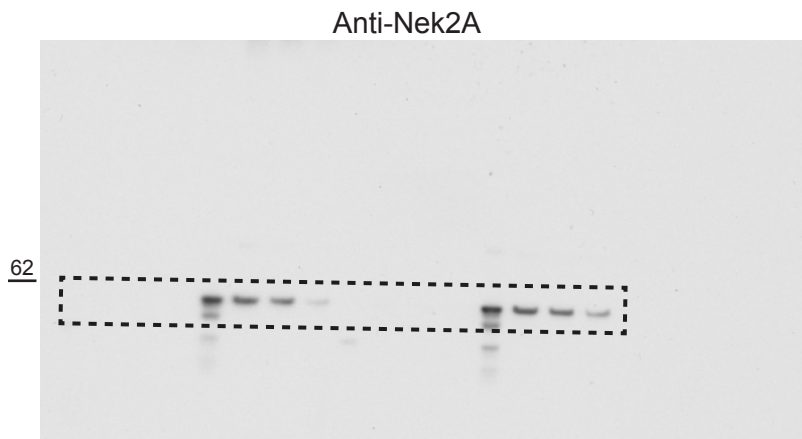

Supplement: Supplementary file 15 — Source Data for Figure 1 [file EMBR-21-e49831-s013.pdf]

**Figure 2**

**C**

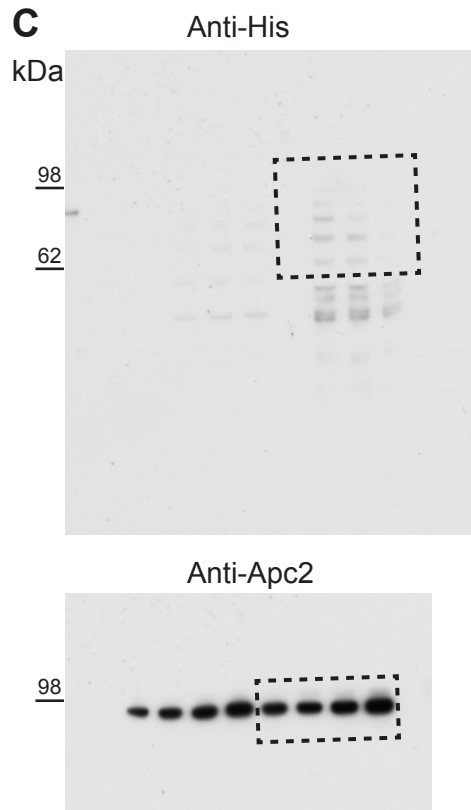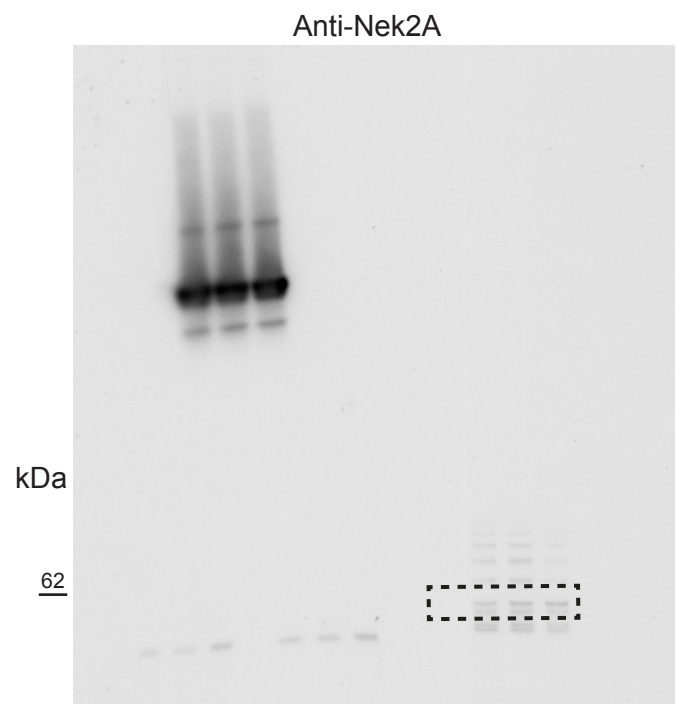

**D**

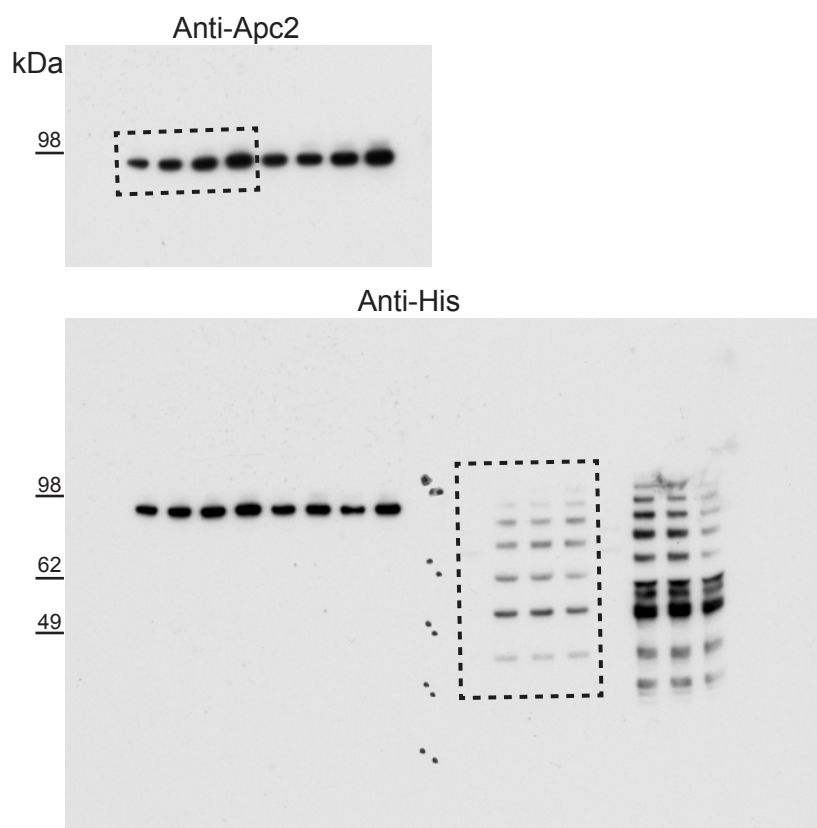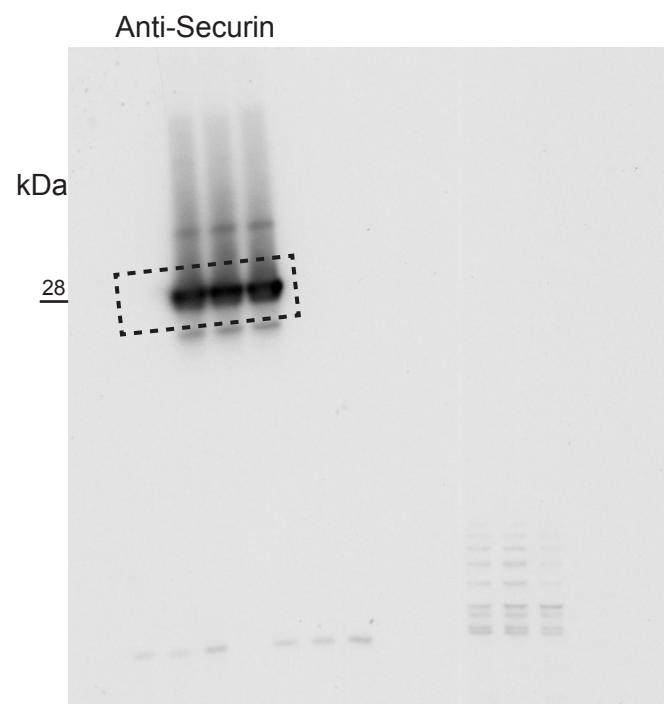

Supplement: Supplementary file 16 — Source Data for Figure 2 [file EMBR-21-e49831-s014.pdf]

Figure 3

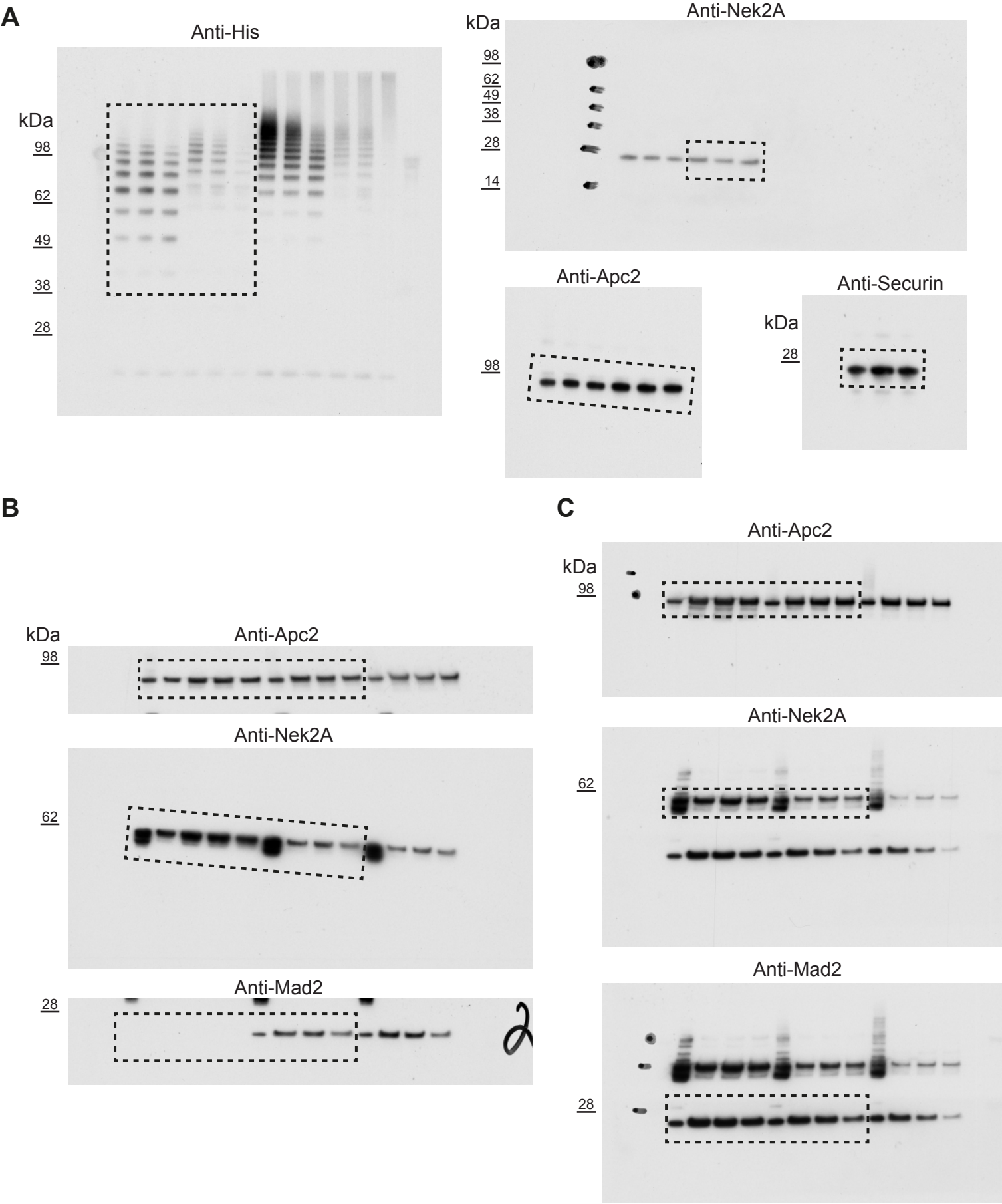

Figure 3

D

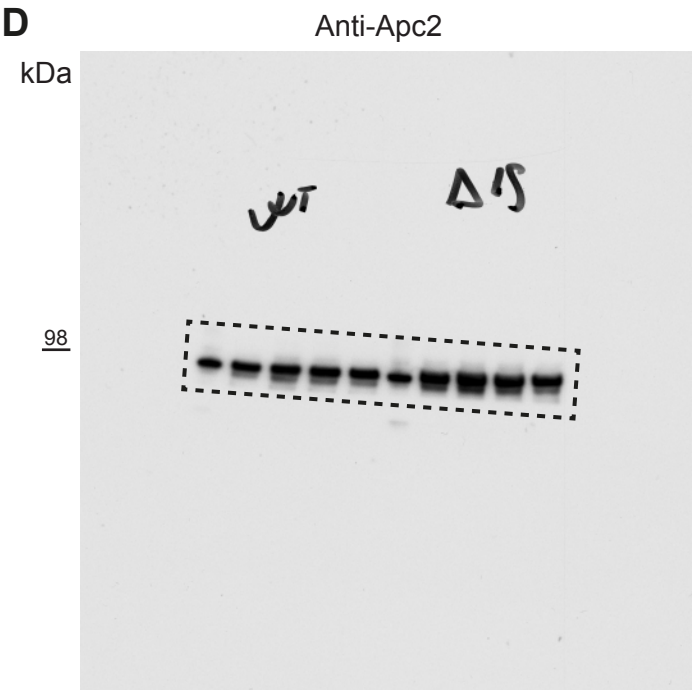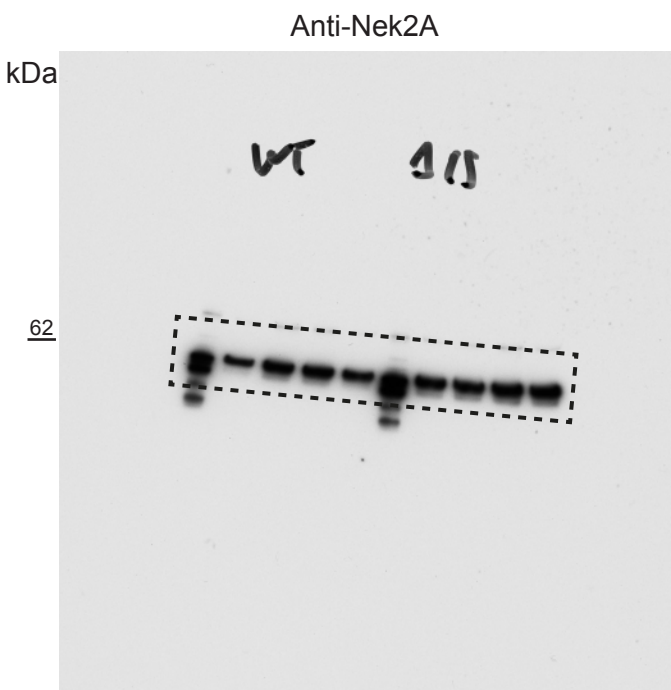

E

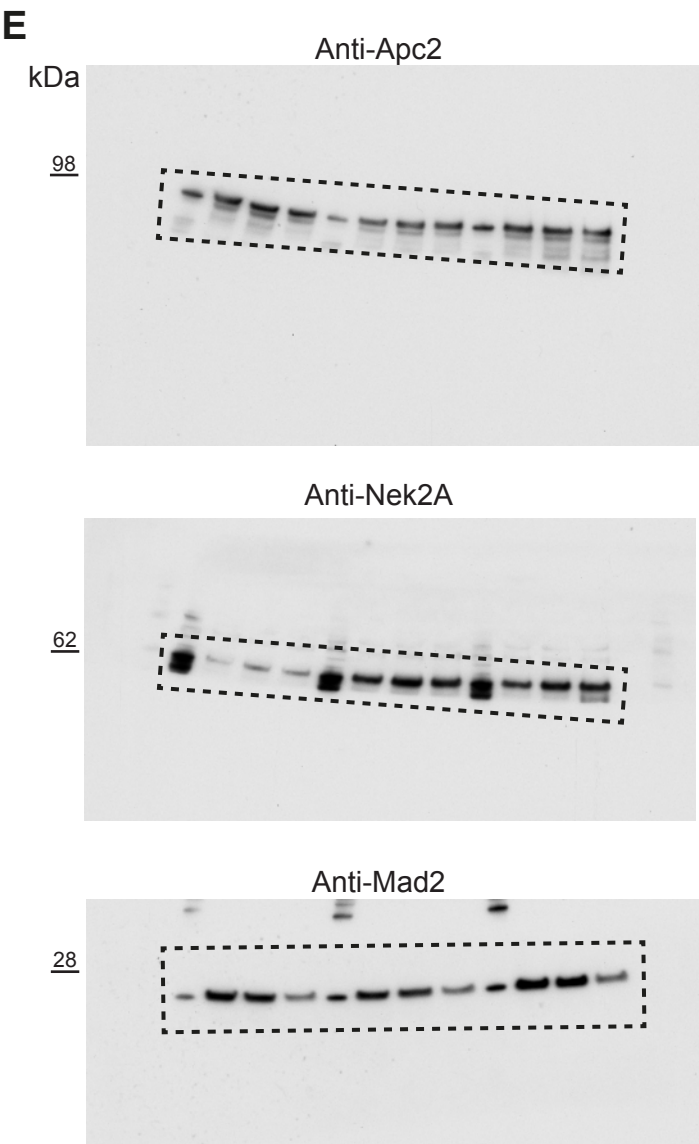

F

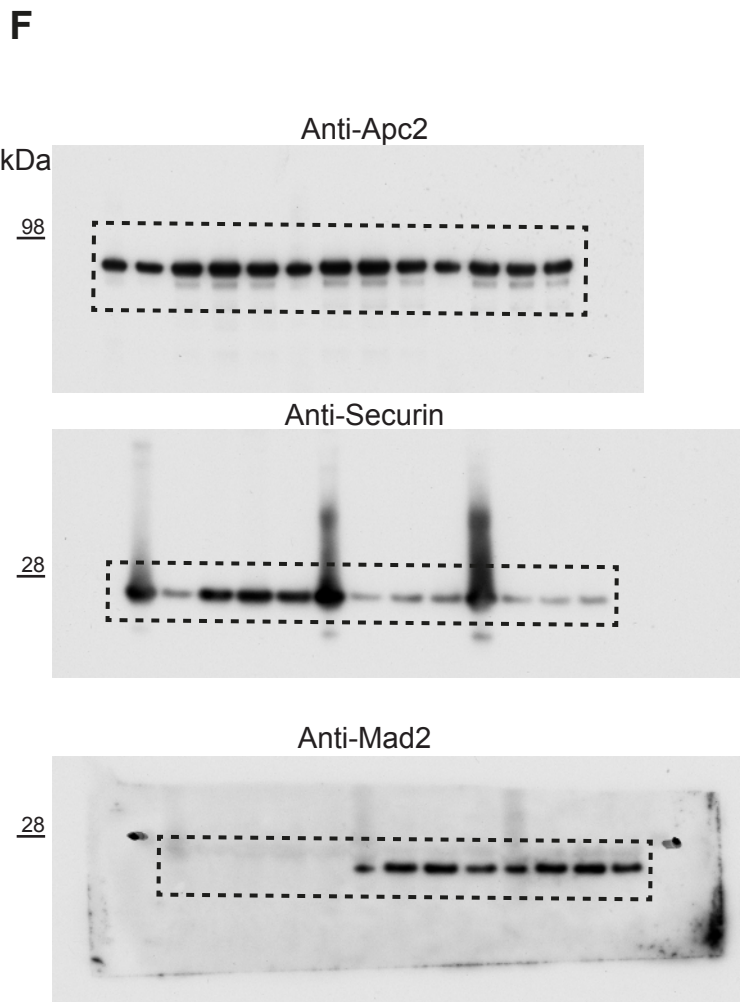

Supplement: Supplementary file 17 — Source Data for Figure 3 [file EMBR-21-e49831-s015.pdf]

Figure 4

B

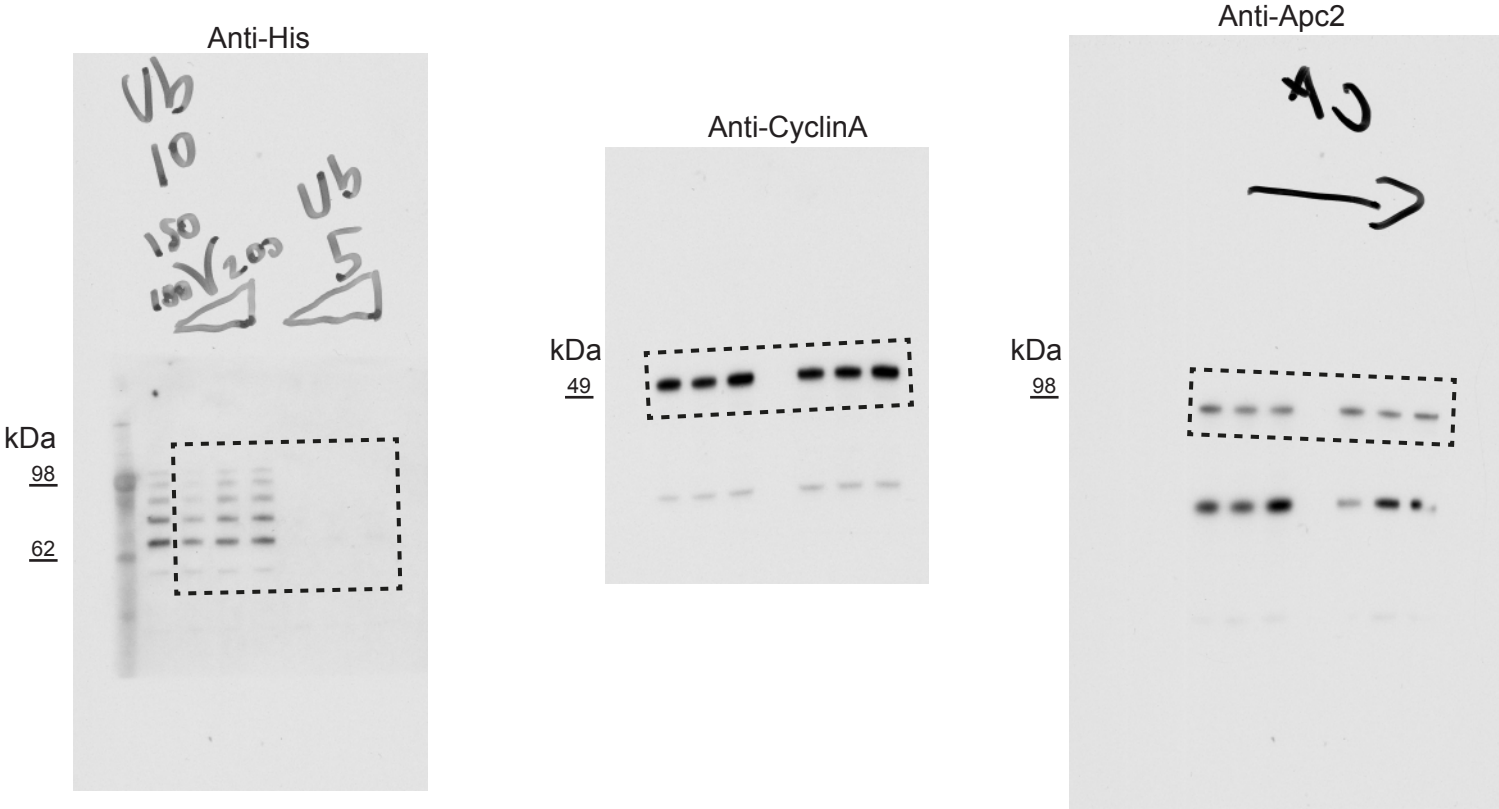

C

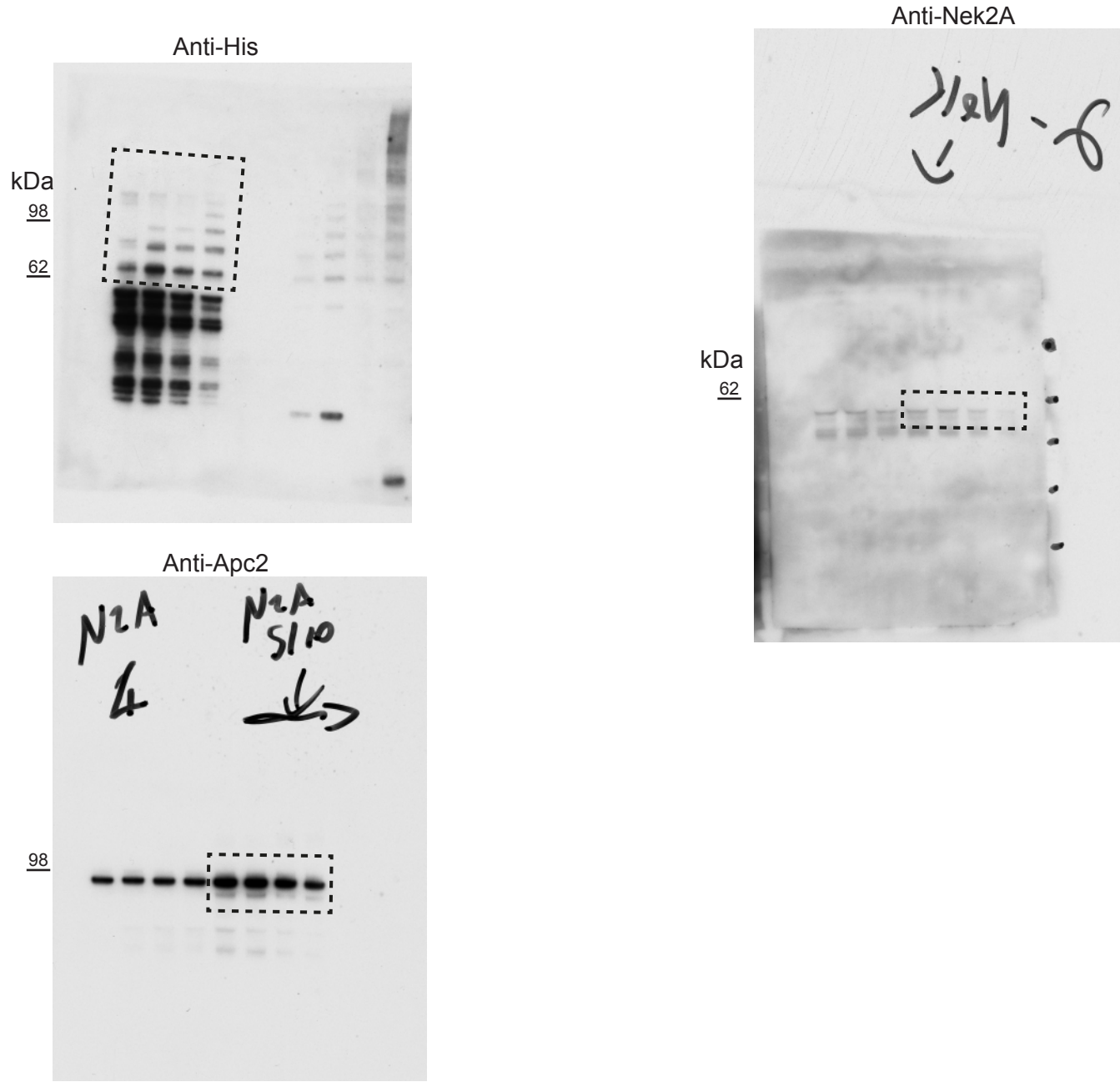

D

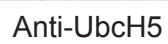

Supplement: Supplementary file 18 — Source Data for Figure 4 [file EMBR-21-e49831-s016.zip › Source data Blots Figure4.pdf]
